# A Ferroptosis-Related Prognostic Signature Based on Antitumor Immunity and Tumor Protein p53 Mutation Exploration for Guiding Treatment in Patients With Head and Neck Squamous Cell Carcinoma

**DOI:** 10.3389/fgene.2021.732211

**Published:** 2021-09-20

**Authors:** Xin Fan, YangShaobo Ou, Huijie Liu, Liangzhen Zhan, Xingrong Zhu, Mingyang Cheng, Qun Li, Dongmei Yin, Lan Liao

**Affiliations:** ^1^The Affiliated Stomatological Hospital of Nanchang University, Nanchang, China; ^2^The Key Laboratory of Oral Biomedicine, Jiangxi Province, Nanchang, China; ^3^Wujing Community Health Service Center, Shanghai, China

**Keywords:** head and neck squamous cell carcinoma, ferroptosis, immune microenvironment, mutation, clinical treatment

## Abstract

**Background:** Due to the lack of accurate guidance of biomarkers, the treatment of head and neck squamous cell carcinoma (HNSCC) has not been ideal. Ferroptosis plays an important role in tumor suppression and treatment of patients. However, tumor protein p53 (TP53) mutation may promote tumor progression through ferroptosis. Therefore, it is particularly important to mine prognostic-related differentially expressed ferroptosis-related genes (PR-DE-FRGs) in HNSCC to construct a prognostic model for accurately guiding clinical treatment.

**Methods:** First, the HNSCC data obtained from The Cancer Genome Atlas (TCGA) was used to identify PR-DE-FRGs for screening candidate genes to construct a prognostic model. We not only used a variety of methods to verify the accuracy of the model for predicting prognosis but also explored the role of ferroptosis in the development of HNSCC from the perspective of the immune microenvironment and mutation. Finally, we explored the correlation between the prognostic model and clinical treatment and drew a high-precision nomogram to predict the prognosis.

**Results:** Seventeen of the 29 PR-DE-FRGs were selected to construct a prognostic model with good predictive performance. Patients in the low-risk group were found to have a greater number of CD8 + T cells, follicular helper T cells, regulatory T cells, mast cells, T-cell costimulations, and type II interferon responses. A higher tumor mutation burden (TMB) was observed in the low-risk group and was associated with a better prognosis. A higher risk score was found in the TP53 mutation group and was associated with a worse prognosis. The risk score is closely related to the expression of immune checkpoint inhibitors (ICIs)-related genes such as PD-L1 and the IC50 of six chemotherapeutic drugs. The nomogram we constructed performs well in predicting prognosis.

**Conclusion:** Ferroptosis may participate in the progression of HNSCC through the immune microenvironment and TP53 mutation. The model we built can be used as an effective predictor of immunotherapy and chemotherapy effects and prognosis of HNSCC patients.

## Introduction

Head and neck squamous cell carcinomas (HNSCCs) are neoplasms affecting different tissues and organs in the head and neck region including the tongue, mouth, nasopharynx, larynx, and throat (Fang et al., 2018). As the sixth most common cancer in the world, head and HNSCC affects more than 700,000 new patients each year, with a mortality rate of approximately 40–50% (Bray et al., 2018; Cohen et al., 2019). Due to the lack of effective early monitoring and screening factors, HNSCC is difficult to be detected in the early stages (Cui et al., 2021). At present, surgery, radiotherapy and chemotherapy based on the location and clinical stage of HNSCC are still identified as commonly used clinical methods for the treatment of HNSCC (Yi L. et al., 2020). However, as one of the core treatment options, recurrence induced during radiotherapy has become the main reason for the failure of HNSCC treatment (He et al., 2019). The treatment that can be used is very limited in view of the poor prognosis of advanced HNSCC. In recent years, the prognosis of patients with advanced malignant tumors has been significantly improved with the clinical application of more and more cancer-corresponding immune checkpoint inhibitors (ICIs). After the anti-PD-1 monoclonal antibodies pembrolizumab and nivolumab were approved for the treatment of recurrent or metastatic HNSCC in 2016, first, ICIs have shown increasing potential in the treatment of HNSCC (Quan et al., 2019). Unfortunately, during HNSCC treatment, ICIs may cause severe morbidity or, in rare cases, mortality, and the objective response rate is only 20% (Wang et al., 2019a; Tada et al., 2020). Therefore, it is urgent to find highly sensitive markers that can predict the benefits of various clinical treatments to significantly reduce the side effects of treatment.

Ferroptosis is a kind of regulatory necrosis driven by iron-dependent phospholipid peroxidation, which is regulated by cell metabolism, redox homeostasis, and various signaling pathways related to cancer (Yi J. et al., 2020). As a non-apoptotic form, the role of ferroptosis in the physiological processes of many diseases, especially its important role in tumor suppression, has been confirmed by many studies (Jiang et al., 2015; Zhang et al., 2018). Recent results indicated that ferroptosis mediates the tumor suppressor activity of interferon gamma secreted by CD8 + T cells in response to immune checkpoint blockade, indicating that the immune system may partly prevent tumorigenesis through ferroptosis (Wang et al., 2019c). Luo et al. (2021) also concluded through meta-analysis that ferroptosis could inhibit tumor growth and contribute to chemotherapy sensitivity. In addition, the activation of ferroptosis has also been proven to help cancer treatments, such as immune checkpoint blockade (Wang et al., 2019c) and radiotherapy (Lei et al., 2020). Ferroptosis induction to achieve cancer cell death may provide a new perspective for the treatment of metastatic cancer and malignant tumors resistant to traditional therapies (Liang et al., 2019; Xia et al., 2020).

As a reflection of the number of mutations in tumor cells, tumor mutation burden (TMB) has been observed to be closely related to the efficacy of ICIS and prognosis in many studies (Devarakonda et al., 2018; Cao et al., 2019; Samstein et al., 2019; Wang et al., 2019c; Wu et al., 2019). As a tumor suppressor gene, missense mutations in the TP53 gene are common in human cancers (Mantovani et al., 2019). In many sporadic cancers, TP53 mutations are associated with poor prognosis (Kandoth et al., 2013). Mutated p53 not only no longer plays the role of suppressing cancer but also assists in the development of cancer by depriving the cell of the tumor suppressor response (Mantovani et al., 2019). In addition to the loss of tumor suppressor function, TP53 mutations also inhibit ferroptosis by altering cellular iron acquisition and metabolism, thereby promoting cancer progression (Thompson et al., 2020). Therefore, it is very likely to find some potential roles of ferroptosis in the development of HNSCC from the perspective of TP53 mutation.

In view of the unsatisfactory treatment methods and lack of ideal models to guide treatment, it is particularly important to explore the ferroptosis-related genes that play a role in HNSCC and construct a new effective prognosis model to predict the prognosis of HNSCC and guide clinical treatment precisely. Moreover, the role of ferroptosis in HNSCC is unclear, and it is urgent to explore the mechanism of ferroptosis from the perspective of immunity and mutation. For this purpose, we used the HNSCC cohort data from The Cancer Genome Atlas (TCGA) to screen the prognostic-related differentially expressed ferroptosis-related genes (PR-DE-FRGs) to construct a prognostic model. We not only used a variety of methods to verify the performance in predicting prognosis of the model but also explored the potential mechanism of the ferroptosis-related model in the development and prognosis of HNSCC from the perspective of immune microenvironment and mutation. Taking into account the close correlation between ferroptosis and clinical treatment, we have further explored the clinical application value of prognostic models, including the guiding value of ICIS treatment, chemotherapy, and the combined model constructed by the prognostic model to predict the prognosis.

In general, our research will provide a model of ferroptosis-related genes with good predictive performance. In addition, this model performs well in clinical applications. It can be used to guide immunotherapy and chemotherapy and predict the prognosis of HNSCC patients. In addition, our research will also explore the possible role of the immune microenvironment and TP53 mutation in the development of HNSCC, providing a new perspective for subsequent research.

## Materials and Methods

### Data Acquisition and Identification of Differentially Expressed Ferroptosis-Related Genes

The RNA-sequencing data in level 3 and corresponding clinical data of 504 HNSCC samples and 44 adjacent normal tissues were downloaded from TCGA database^1^ for the analysis on February 3, 2021. In addition, somatic gene mutation and the copy number variations (CNVs) data on TCGA also have been downloaded. The lists of FRGs used in our analysis were derived from the FerrDb Database,^2^ which is the first database known for the manual collection and management of ferroptosis-related markers and regulatory factors and ferroptosis-related diseases in the world. A total of 258 FRGs from 784 articles on ferroptosis in PubMed database were included in FerrDb. The annotation of these genes revealed 108 driver genes, 69 suppressor genes, and 111 gene markers. In view of the fact that these data were both publicly available in TCGA, the approval of the local ethics committee was exempted for this research. The gene expression quantification of the same 246 FRGs between the lists of FRGs and RNA-sequencing data of HNSCC samples from TCGA was extracted. R package limma was used for differential expression analysis between 504 HNSCC samples and 44 adjacent normal tissues to identify the DE-FRGs. Since there were fewer FRGs, our analysis set the filter conditions to | log2 fold change| (| log2FC| > 0) along with false discovery rate <0.01.

### Construction of Regulatory Networks and Prognostic Model Based on Prognostic Differentially Expressed Ferroptosis-Related Genes

We integrated data from TCGA to obtain the same samples that both held complete overall survival (OS) and messenger RNA (mRNA) expression data. Univariate Cox analysis with the cutoff value of *p* < 0.05 was performed to select FRGs with prognostic values. After the minimum required interaction score was set at medium confidence (0.400), protein–protein interaction (PPI) network consisting of 29 prognostic DE-FRGs was generated in the STRING database, which was widely known for searching known protein interaction relationships online. In addition, R package reshape2, psych, RColorBrewer, and igraph were used for correlation analysis of 29 genes and visualization of related networks. A total of 499 samples with complete OS data and mRNA expression value of prognostic DE-FRGs were randomly matched to the training set (*n* = 300) and the test set (*n* = 199) at a ratio of 6:4. The expression values of 29 prognostic DE-FRGs from the training group were used in the least absolute shrinkage and selection operator (LASSO) regression analysis, which could screen out highly related genes and minimize the risk of overfitting for screening signatures to accurately forecast the clinical efficacy in HNSCC cases. Finally, a prognostic model based on 17 DE-FRGs was constructed by selecting the optimal penalty parameter, which was determined by the minimum 10-fold cross-validation. Next, we used the coefficients obtained by the LASSO regression algorithm in the following risk score equation: risk score = sum of corresponding coefficients × its gene’s expression value (Gao et al., 2010). In addition, Kaplan–Meier survival curve was used to analyze the relationship between expression of each gene in the model and OS.

### Validation of the Prognostic Model

The training set, test set, and whole set were used to evaluate the predictive power of the prognostic model simultaneously. However, three sets obtained from the TCGA data were from a single cohort. The risk score of each case that would be divided into high- and low-risk groups according to the median value of their risk scores was calculated separately in the training set, test set, and whole set. As soon as risk score was obtained, we used the R tool to visualize the specific risk score value and survival status of each sample in each set in the model. R package survival and survminer were used to perform Kaplan–Meier to verify the performance of the model to distinguish the survival of HNSCC patients. The multifactor receiver operating characteristic (ROC) curves were used not only to verify the accuracy of the model but also to optimality of the model compared to other factors in predicting survival. We also performed principal component analysis (PCA) with prcomp function of the R package stats based on the expression of genes in each set. In addition, univariate and multivariate cox regression analysis were performed on the variables (age, gender, grade, stage, T, N, and risk score) from three sets to determine whether the risk score could be used as an independent prognostic predictor of OS.

### Gene Ontology and Kyoto Encyclopedia of Genes and Genomes Enrichment Analysis

The R package limma was utilized to determine the differential genes (DEGs) between the high- and low-risk group among the three sets based on the filter condition (| log2FC | ≥ 1, FDR < 0.05). We used R package clusterProfiler to run Kyoto Encyclopedia of Genes and Genomes (KEGG) and Gene Ontology (GO) to elucidate which functions and pathways of these DEGs are enriched.

### The Relationship Between Prognostic Model Immune Cells/Immune Functions

To further reveal the correlation between the prognostic model and immune tumor immune microenvironment, the single-sample gene set enrichment analysis based on R packages GSEABase and gsva was used to quantify the scores of immune cells and functions. The difference analysis of the scores of immune cells and functions between different risk groups based on the prognostic model were performed to draw a box plot.

Since ferroptosis is closely related to cancer immunity, we also downloaded the data of the content of T cells CD8 + and regulatory T cells (Tregs) in each HNSCC sample calculated by six advanced algorithms from the TIMER 2.0 database,^3^ including TIMER, MCPCOUNTER, CIBERSORT, XCELL, QUANTISEQ, and EPIC. In further analysis, we separately analyzed the correlation between the content of these cells and the PR-DE-FRGs used in constructing the model.

### Mutation and Copy Number Variation Analysis

We downloaded the somatic gene mutation data and corresponding clinical data of HNSCC samples in the TCGA dataset. These mutation data were used to evaluate the relationship between the mutation and the prognostic model of HNSCC patients who owned available mutation data. After using VarScan to detect the mutation annotation format (MAF) files of somatic mutations in HNSCC samples, the R package GenVisR was used to visualize the 30 most frequently mutated genes in the high- and low-risk groups, respectively, (Koboldt et al., 2009). The R package maftools was used to calculate the TMB who was defined as the number of somatic, coding, indels mutations, and base substitutions in a million bases of the genome. To explore the influences of TMB on survival, Kaplan–Meier (KM) analysis was used to compare the differences in survival between high- and low-TMB groups. According to the TP53 mutation status, the TCGA samples were assigned to the wild group and the mutant group. To explore the association between ferroptosis and TP53 mutation, we compared the difference in risk scores between the TP53 mutation group and TP53 wild group. Not only that, KM analysis was used to compare the difference in OS. We extracted the CNV data of 16 genes used to build the model in 526 HNSCC samples. After statistics on the CNV frequency of these genes, the corresponding results were visualized. The locations of CNV changes in these 16 genes on the chromosome were also visualized in the circle graph.

### Correlation Between Prognostic Model and Clinical Treatment

The immunophenoscore (IPS) data of HNSCC patients were obtained from The Cancer Immunome Atlas (TCIA) database.^4^ The patient’s IPS was obtained by evaluating the gene expression of the four cell types (effector cells, immunosuppressive cells, MHC molecules, and immunomodulators) that determine immunogenicity (Liu et al., 2020). As the IPS score increases, the immunogenicity increases (Liu et al., 2020). To analyze the potential correlation between prognostic models and immunotherapy, Spearman correlation analysis was run to evaluate the correlation between risk score and four types of IPS and the expression of eight ICIs-related genes (CD274, CD40,CTLA4, PDCD1, CD96, TIGIT, LAG3, and HAVCR2), respectively. The results of the correlation were visualized in the form of a lollipop graph through the R package ggplot2. To predict the sensitivity of HNSCC patients to chemotherapy drugs recommended by National Comprehensive Cancer Network (NCCN) guidelines in the clinic, the R package pRRophetic was used to predict the half maximal inhibitory concentration (IC50) of different risk sample to chemotherapy drugs. The IC50 difference between high- and low-risk populations was compared. The cell line expression data in Cancer Drug Sensitivity Genomics (GDSC) database and RNA sequencing transcriptome data in TCGA database were used to construct the ridge regression model to predict the IC50 of the drug in this R package (Geeleher et al., 2014).

### Construction and Evaluation of Nomogram

To create a more clinically applicable quantitative tool for predicting 1-, 2-, and 3-year OS of HNSCC patients, the factors that may affect survival containing risk scores and clinical risk factors were used to construct a multivariate cox regression model by the R package rms to draw nomogram. The calibration curve was then used to evaluate the accuracy of survival prediction in the nomogram. In addition, we not only drew the ROC curves in 1, 2, and 3 years of nomogram but also compared ROC curves of nomogram with risk score and other clinical factors to verify the optimality of nomograph in predicting prognosis.

### Immunohistochemical Staining

In order to further verify the differential expression of the 17 PR-DE-FRGs used to construct the model between HNSCC tissues and normal tissues of the head and neck, we used the result of immunohistochemistry (IHC) staining from the human protein atlas database.^5^ Finally, the immunohistochemical images of the protein expression of 15 PR-DE-FRGs in HNSCC tissues and normal tissues of the head and neck were obtained.

### Statistical Analysis

According to the distribution characteristics, Student’s *t*-test or Mann–Whitney test was used to compare the difference between continuous variables, while the chi-square test or Fisher exact test was used to compare the difference between categorical variables. Univariate Cox regression analysis was used to confirm DE-FRGs related to prognosis, which would be screened to construct the optimal prognostic model by LASSO regression. The nomogram was constructed using multivariate Cox regression. Kaplan–Meier curve with log-rank test was utilized to compare survival between different groups. The prognostic ability of each factor for prognosis was evaluated by ROC curve. The independent predictive value of risk scores of prognostic model for survival was evaluated with univariate and multivariate Cox regression analyses. All analyses in our study were performed in R programming language (version 4.0.3) and SPSS Statistics software 22.

## Results

### Identification of Differentially Expressed Ferroptosis-Related Genes in Head and Neck Squamous Cell Carcinoma

**FIGURE 1:**
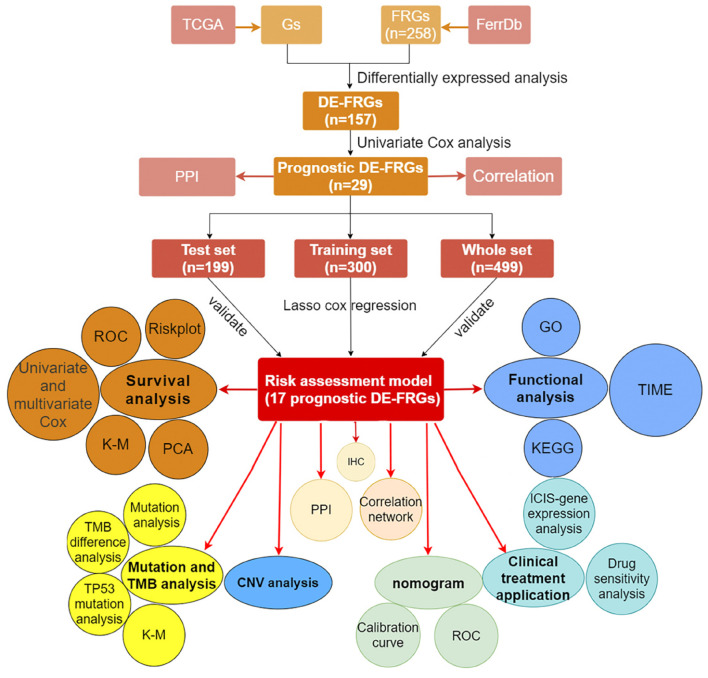
The workflow of research methods.

**FIGURE 2:**
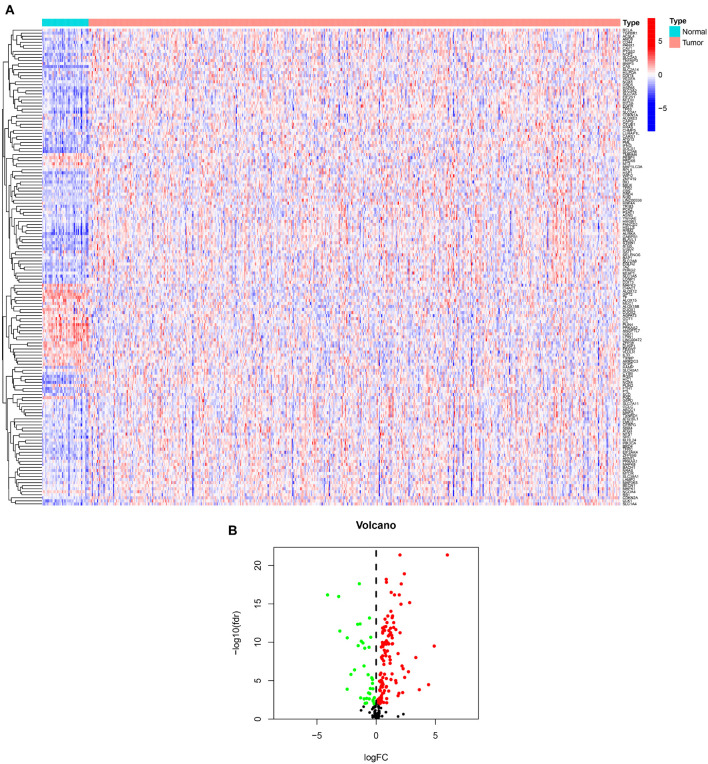
Overview of DE-FRGs. **(A)** The heat map shows the expression profile of DE-FRGs. **(B)** The volcano chart shows the regulation profile of DE-FRG. Red dots represent upregulated genes, while green dots represent downregulated genes.

We showed the workflow of this study in Figure 1. In the first, gene transfer format files from ensembl were used to annotate the data of 504 HNSCC and 44 adjacent normal tissues. The gene expression quantification of 246 FRGs were extracted to identify 157 DE-FRGs (Figure 2A). Among 157 FRGs, 36 genes were identified as downregulated and 121 genes were identified as upregulated (Figure 2B).

### Construction of Regulatory Networks and Prognostic Model Based on Prognostic Differentially Expressed Ferroptosis-Related

**FIGURE 3:**
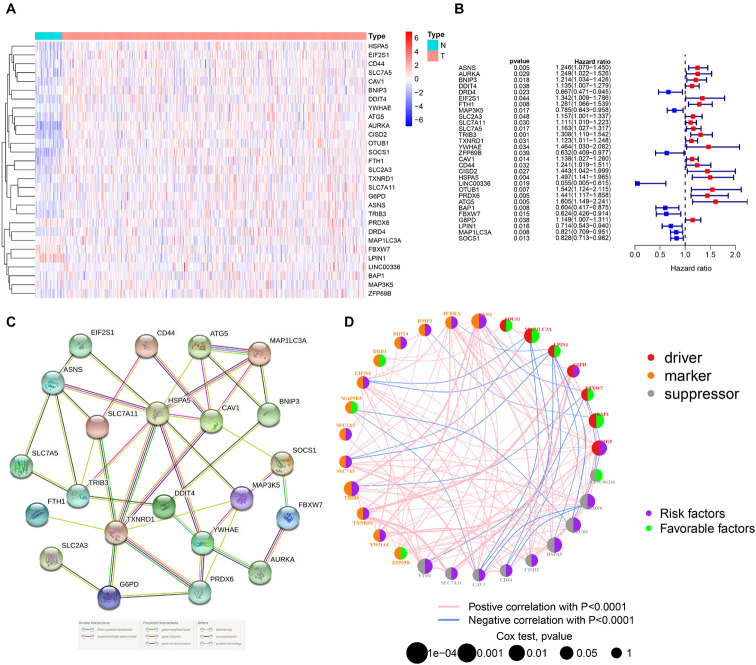
Identification of PR-DE-FRGs used to construct association networks. **(A)** The heatmap used to show the expression profile of 29 PR-DE-FRGs. **(B)** The forest plot used to show the results of univariate Cox regression analysis between 29 PR-DE-FRGs and survival. **(C)** PPI of 29 PR-DE-FRGs. The bottom of the figure shows that the different colored lines between nodes represent different sources of evidence. **(D)** Correlation network of 29 PR-DE-FRGs. The left and right halves of the circle represent the attributes of the gene and its influence on the prognosis, respectively.

**FIGURE 4:**
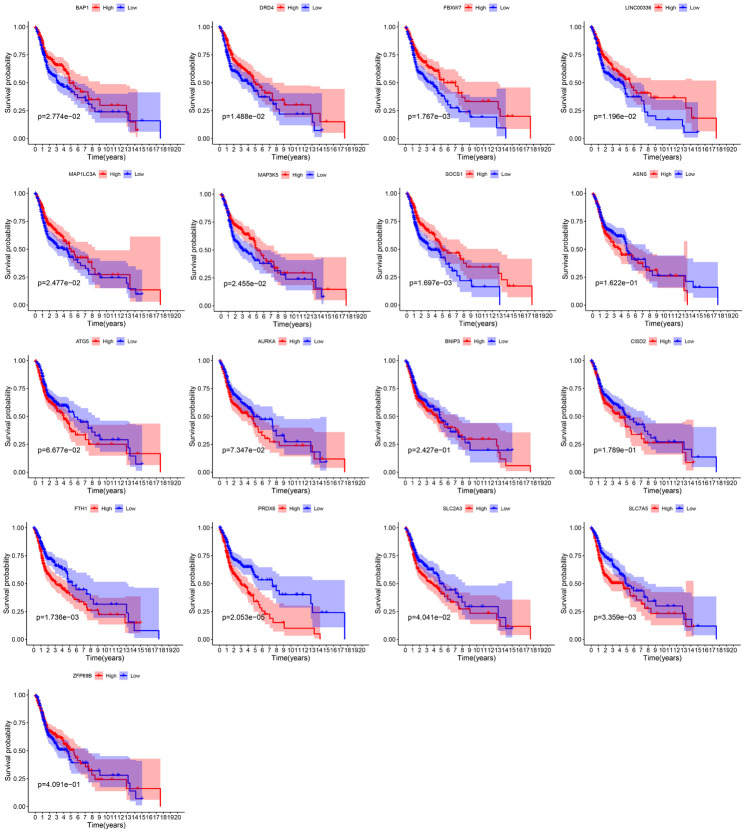
Survival curves of 17 PR-DE-FRGs used to construct the model.

By integrating the mRNA expression and clinical data of HNSCC patients in TCGA set, we obtained 499 samples whose clinical characteristics are shown in Table 1. Twenty-nine prognostic DE-FRGs were screened out by univariate Cox analysis of OS (Figures 3A,B). Seven hub genes, namely, HSPA5, CAV1, TXNRD1, ASNS, TRIB3, TWHAE, and MAP3K5, were indicated in the interaction network consisting of 29 prognostic DE-FRGs (Figure 3C). Figure 3D shows the correlation between 29FRGs. Next, 17 DE-FRGs, namely, ASNS, AURKA, BNIP3, DRD4, FTH1, MAP3K5, SLC2A3, SLC7A5, ZFP69B, CISD2, LINC00336, PRDX6, ATG5, BAP1, FBXW7, MAP1LC3A, and SOCS1, were determined based on the optimal value of λ by the LASSO regression analysis, which used the data from the training set for further analysis. Finally, a prognostic model was established, whose equation was as follows: risk score = (0.0538^∗^ASNS expression value) + (0.0513^∗^AURKA) + (0.0425^∗^BNIP3) + (−0.0545^∗^DRD4) + (0.0209^∗^FTH1) + (−0.0849^∗^MAP3K5) + (0.1317^∗^SLC2A3) + (0.0561^∗^SLC7A5) + (−0.6544^∗^ZFP69B) + (0.1769^∗^CISD2) + (−0.2734^∗^LINC00336) + (0.0458^∗^PRDX6) + (0.3976^∗^ATG5) + (−0.1081^∗^BAP1) + (−0.0676^∗^FBXW7) + (−0.0917^∗^MAP1LC3A) + (−0.1629^∗^SOCS1). ASNS, AURKA, BNIP3, FTH1, SLC2A3, SLC7A5, CISD2, ATG5, and PRDX6 (HR > 1) were identified as risk factors, while DRD4, MAP3K5, ZFP69B, LINC00336, BAP1, FBXW7, MAP1LC3A, and SOCS1 (HR < 1) were confirmed protective factors for prognosis (Figure 3B). In addition, better OS was observed in the high expression of BAP1, DRD4, FBXW7, LINC00336, MAP1LC3A, MAP3K5, and SOCS1 and the low expression of FTH1, PRDX6, SLC2A3, and SLC7A5 by the Kaplan–Meier survival in Figure 4 (*p* < 0.05).

**TABLE 1:** Clinical characteristics of the HNSCC samples in training, test, and whole sets.

	Training set (*n* = 300)	Test set (*n* = 199)	Whole set (*n* = 499)
**Gender (%)**			
Male	218 (72.7%)	154 (22.6%)	366 (73.3%)
Female	88 (29.3%)	45 (22.6%)	133 (26.7%)
**Age (median, range)**	61 (24–87)	61 (19–90)	61 (19–90)
**Survival status**			
OS-days (median, range)	593 (14–6,417)	491 (1–5,252)	568 (1–6,417)
OS-state [alive(%)/dead(%)]	187 (62.3%)/113 (36.7%)	117 (58.8%)/82 (41.2%)	304 (60.9%)/195(39.1%)
**Grade (%)**			
Grade 1–2	221 (73.7%)	138 (69.3%)	359 (71.9%)
Grade 3–4	65 (21.7%)	56 (28.1%)	121 (24.3%)
Unknown	14 (4.6%)	5 (2.6%)	19 (3.8%)
**Stage (%)**			
I–II	61 (20.3%)	33 (16.6%)	94 (18.8%)
III–IV	192 (64.0%)	145 (72.9%)	337 (67.6%)
Unknown	47 (15.7%)	21 (10.6%)	68 (13.6%)
**T (%)**			
0–2	108 (36.0%)	69 (34.7%)	177 (35.5%)
3–4	152 (50.7%)	115 (57.8%)	267 (53.5%)
Unknown	40 (13.3%)	15 (7.5%)	55 (11.0%)
**M (%)**			
0	104 (34.7%)	82 (41.2%)	185 (37.1%)
1	1 (0.3%)	0 (0.0%)	1 (0.2%)
Unknown	195 (65.0%)	117 (58.8%)	313 (62.7%)
***N* (%)**			
0–1	141 (47.0%)	94 (47.2%)	235 (47.1%)
2–3	104 (34.7%)	68 (34.2%)	171 (34.3%)
Unknown	55 (18.3%)	37 (18.6%)	93 (18.6%)

### Validation of Prognostic Model

**FIGURE 5:**
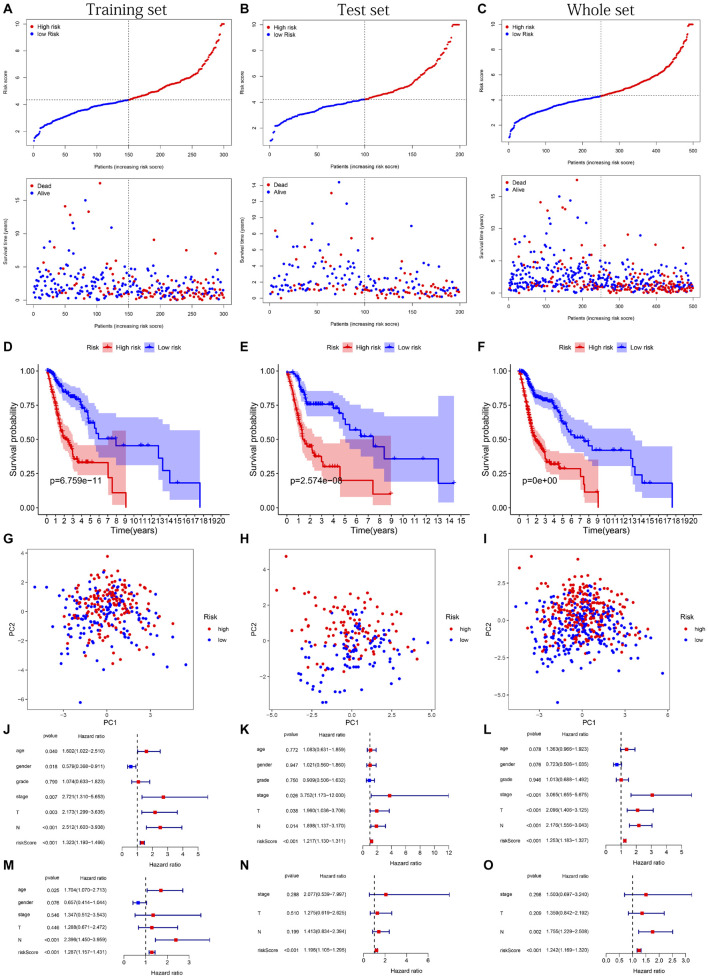
Risk plots, survival curves, and PCA discrete trend charts based on prognostic model and forest plots based on univariate and multivariate Cox regression analysis results. **(A–C)** Risk plots of training, test, and whole sets, respectively. **(D–F)** Survival curves of training, test, and whole sets, respectively. **(G–I)** PCA discrete trend charts of training, test, and whole sets, respectively. **(J–L)** The forest plots show the results of the univariate Cox regression analysis of the training, test, and whole sets, respectively. **(M–O)** The forest plots show the results of the multivariate Cox regression analysis of the training, test, and whole sets, respectively.

**FIGURE 6:**
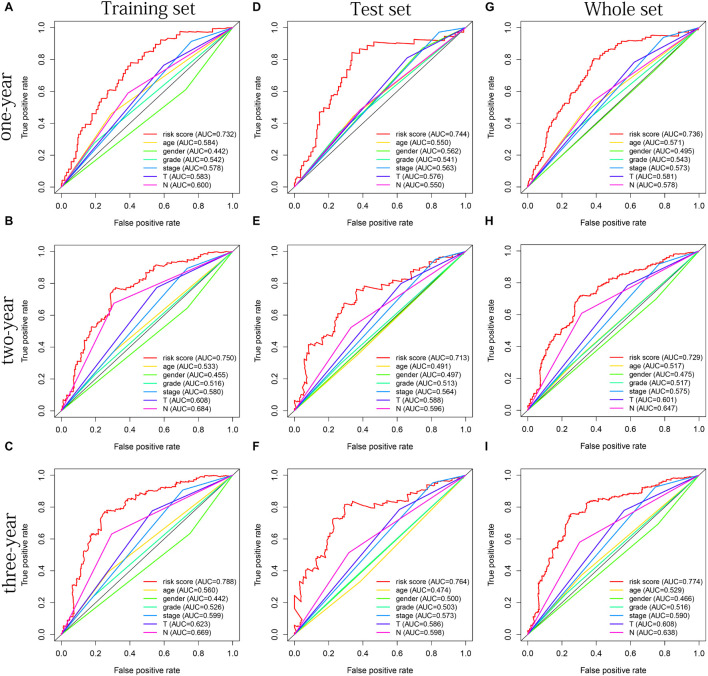
The multifactor ROC curves confirms the best predictive performance of models. **(A–C)** One, 2, and 3 years of multifactor ROC curves based on training set, respectively. **(D–F)** One, 2, and 3 years of multifactor ROC curves based on test set, respectively. **(G–I)** One, 2, and 3 years of multifactor ROC curves based on whole set, respectively.

The distribution of risk scores, OS, and OS status of 17 signatures in training, test, and whole sets, which all indicate that as the risk score increases, there is an increasing trend of deaths, is shown in Figures 5A–C. After Kaplan–Meier analysis, we all found that patients in the high-risk group dramatically had lower survival probability in three sets (Figures 5D–F). Figures 5G–I showed the PCA results of the three sets respectively. The univariate COX regression analysis showed that the risk score of the training, test, and whole sets were all significantly correlated with OS (HR = 1.323, 95% CI = 1.193–1.466, *p* < 0.001; HR = 1.217, 95% CI = 1.130–1.311, *p* < 0.001; HR = 1.253, 95% CI = 1.183–1.327, *p* < 0.001) (Figures 5J–L). Risk score was still identified as an independent predictor of OS (HR = 1.287, 95% CI = 1.157–1.431, *p* < 0.001; HR = 1.196, 95% CI = 1.105–1.295, *p* < 0.001; HR = 1.242, 95% CI = 1.169–1.320, *p* < 0.001) in each set by the multivariate Cox regression analysis after adjusting for other clinical confounding factors (Figures 5M–O). To verify the optimality of the model, we drew ROC curve based on the training, test, and whole sets. Among the 3 years, the results in each set showed that all the area under the curve (AUC) values are above 0.7 (Figures 6A–I), which means that this model has better predictive value. It was worth mentioning that consistent results were obtained in the three sets: the risk scores have the largest AUCs of 1, 2, and 3 years in all factors, which indicates that the risk model has the best predictive effect (training set, Figures 6A–C; test set, Figures 6D–F; whole set, Figures 6G–I).

### Gene Ontology and Kyoto Encyclopedia of Genes and Genomes Enrichment Analysis

**FIGURE 7:**
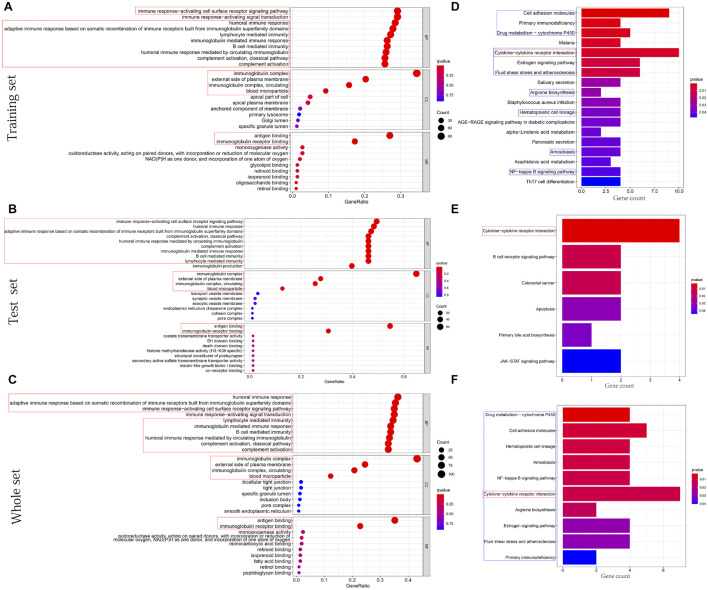
Functions and pathways enrichment DEGs participated in. **(A–C)** The bubble chart, respectively, shows the BPs, CCs, and MFs enriched by GO in training, test, and whole sets. **(D–F)** The histogram, respectively, shows the pathways enriched by KEGG in training, test, and whole sets. The size of the circle represents the number of DEGs enriched on each GO. The color of the circle or cuboid represents the significance of enrichment.

The cellular components (CCs), molecular functions (MFs), and biological processes (BPs) enriched in training, test, and whole sets are shown in Figures 7A–C, respectively. Surprisingly, BPs, MFs, and CCs, which were enriched in all three sets, were almost all immune related, such as immune response-activating cell surface receptor signaling pathway, humoral immune response, adaptive immune response based on somatic recombination of immune receptors built from immunoglobulin superfamily domains, lymphocyte-mediated immunity, immunoglobulin-mediated immune response, B-cell-mediated immunity, humoral immune response mediated by circulating immunoglobulin, complement activation classical pathway, complement activation, antigen binding, immunoglobulin receptor binding, external side of plasma membrane, immunoglobulin complex, immunoglobulin complex circulating, and blood microparticle. After KEGG pathway analysis, it was found that the cytokine–cytokine receptor interaction pathway was enriched in all sets. Figures 7D–F, respectively, showed all results of KEGG pathway for training, test, and whole sets. The blue rectangles in Figures 7D,F also marked the 10 same pathways enriched in training and whole sets.

### The Immune Infiltrating Cells/Immune Functions Content Associated With Prognostic Model

**FIGURE 8:**
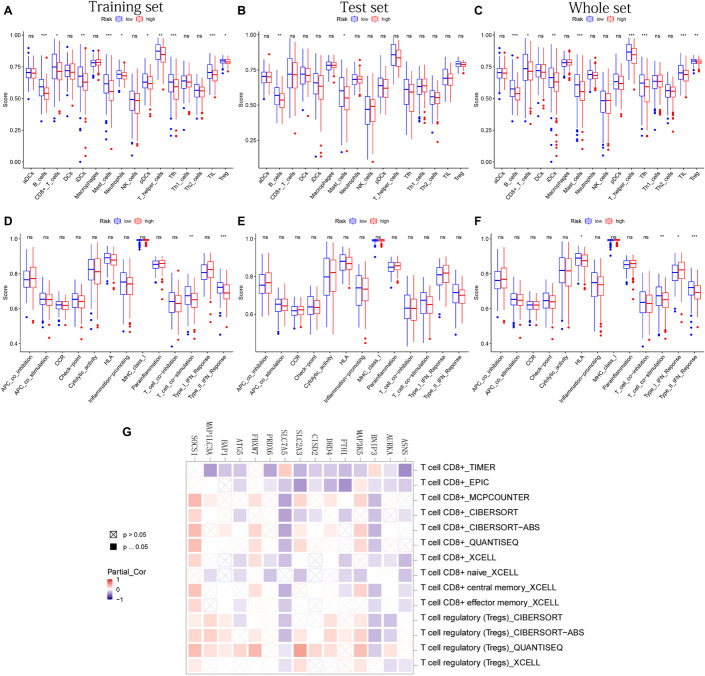
The immune infiltrating cells/immune functions content associated with prognostic model. **(A–C)** The histograms, respectively, show the difference of the immune-infiltrating cells in the training, test, and whole sets, respectively. **(D–F)** The histograms, respectively, show the difference of the immune function in the training, test, and whole sets. The symbol above the histogram shows the significance of the difference. *p < 0.05; **p < 0.01; ***p < 0.001; ns, no significance. **(G)** The correlation between the expression of 15 PR-DE-FRGs used to construct the model and CD8 + T cells/Tregs.

After difference analysis of the scores of 16 immune cells between different risk groups, we found that the score of B cells and mast cells were all significantly higher in the low-risk group in training, test, and whole sets (adjusted *p* < 0.05, Figures 8A–C). It was worth mentioning that the scores of B cells statistically differed between the high- and low-risk groups (adjusted *p* < 0.001, Figures 8A–C), which was closely related with B-cell-mediated immunity enriched significantly in the GO analysis.

In addition, the scores of CD8 + T cells, immature dendritic cells (iDCs), T-helper cells, follicular helper T cells (Tfh), tumor-infiltrating lymphocyte (TIL), and regulatory T cells (Tregs) in the low-risk group all were significantly higher than those in the high-risk group in training and whole set (Figures 8A,C). In terms of 13 immune functions, in both the training and whole sets, it was worth mentioning that the scores of T-cell costimulation and type II IFN response were both observed to be significantly higher in the low-risk groups (Figures 8D,F). Figure 8E shows the difference analysis results of immune functions based on the test set data.

Finally, in addition to ZFP69B and LINC00336, the correlation results of the remaining 15 PR-DE-FRGs with T cells CD8 + /Tregs content were obtained and shown in Figure 8G. Through a comprehensive analysis of the results obtained by multiple calculation methods, it is shown that the expression of ASNS, BNIP3, FTH1, CISD2, SLC7A5, and ATG5 is negatively correlated with the content of CD8 + T cells, whereas the expression of MAP3K5, FBXW7, and SOCS1 is positively correlated with the content of CD8 + T cells. In addition, from the correlation graph, the content of Tregs was not only found to be significantly and negatively correlated with the expression of AURKA, BNIP3, and SLC7A5 but also was found to be significantly and positively correlated with the expression of MAP3K5, DRD4, SLC2A3, BAP1, FBXW7, MAP1LC3A, and SOCS1.

### Mutation Analysis and Copy Number Variation Analysis

**FIGURE 9:**
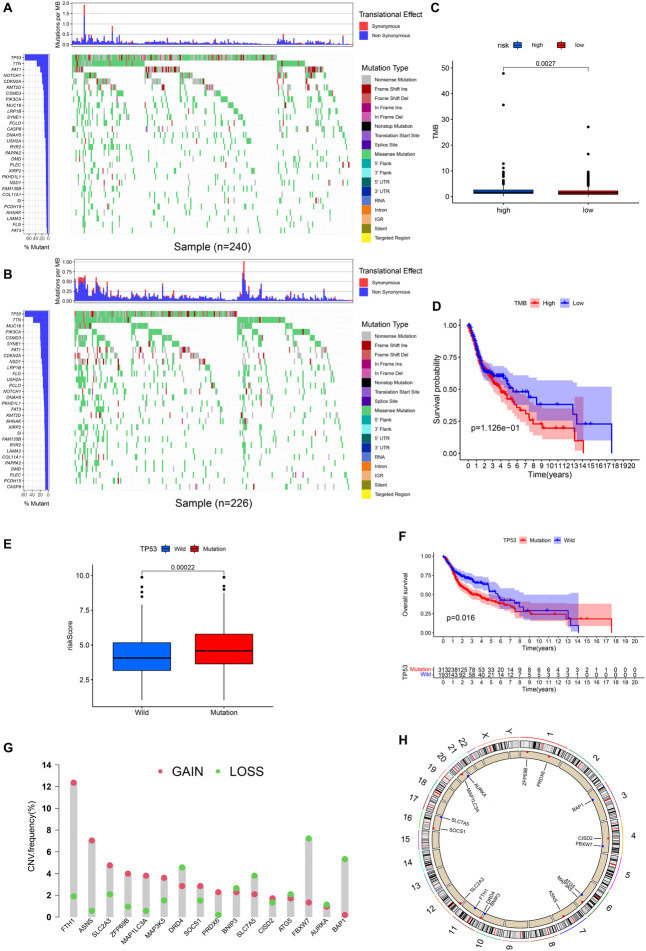
Landscape of Mutation and CNV (copy number variations, CNVs). **(A)** The waterfall chart shows the mutation distribution of patients in the high-risk group. **(B)** The waterfall chart shows the mutation distribution of patients in the low-risk group. The left panel of the waterfall plot shows the mutation frequency, and the genes are sorted by their mutation frequency. The figure on the right shows the type of mutation. The histogram above is the statistical data of TMB of each sample. **(C)** TMB difference between high- and low-risk groups. **(D)** Survival difference curve of patients between high- and low-TMB groups. **(E)** Risk score difference between TP53 mutation group and TP53 wild group. **(F)** Survival difference curve of patients between TP53 mutation group and TP53 wild groups. **(G)** CNV profile of 17 PR-DE-FRGs. The blue dots represent the loss of copy number; the pink dots represent the gain of copy number. **(H)** Circle graph shows the location of comprehensive CNV status of 17 PR-DE-FRGs on 23 chromosomes. The blue dot represents the loss of overall copy number, while the red represents the gain in overall copy number.

Figures 9A,B, respectively, show the mutations of the top 30 most frequent genes of 240 samples in high-risk group and 226 samples in the low-risk group. In the box plot (Figure 9C), the TMB of patients in the low-risk group was shown to be significantly lower. It was observed that OS of samples with low TMB were better in further survival analysis (Figure 9D). Patients in the TP53 mutation group were found to have a higher risk score (Figure 9E). In addition, patients in the TP53 mutation group were observed to have a worse prognosis (Figure 9F). From Figure 9G, we observed that CNVs were common in these 16 genes. Except for BNIP3, SLC7A5, ATG5, FBXW7, AURKA, and BAP1, other genes have a higher frequency of acquiring CNV (Figure 9G). The corresponding locations of these 16 genes on the chromosome and the synthesis of CNV are shown in Figure 9H.

### Clinical Treatment Application of Prognostic Model

**FIGURE 10:**
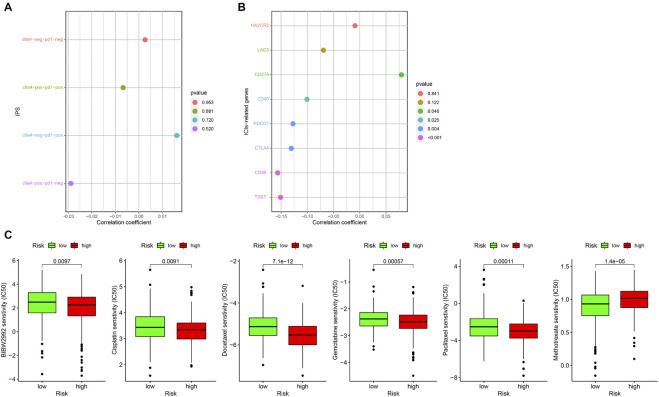
Correlation between prognostic model and clinical treatment. **(A)** The correlation point graph shows the correlation between four types of IPS and risk score. **(B)** The lollipop graph shows the correlation between eight ICIs-related genes and risk score. **(C)** The box diagram shows the difference in IC50 of the six chemotherapy in the high- and low-risk groups.

At present, ICIs such as PD-1 inhibitor pembrolizumab was being used clinically as the first-line treatment of relapsed HNSCC. Unfortunately, none of the four combinations of IPS showed a significant correlation with the risk score (Figure 10A). However, we found that the expression of CD274 (PD-L1) was significantly positively correlated with the risk score, while that of CD40, PDCD1 (PD-1), CTLA4, CD96, and TIGIT were significantly negatively correlated with risk score (all *p* < 0.05; Figure 10B). This result suggests that our model may be used to predict the expression of PD-L1 in patients to predict the effect of immunotherapy. We predicted IC50 of each sample of six chemotherapy drugs (cisplatin, paclitaxel, docetaxel, gemcitabine, methotrexate, and BIBW2992) recommended by NCCN guidelines for HNSCC. It was found that except for methotrexate, the IC50 of the other five chemotherapy drugs in the low-risk group was higher (all *p* < 0.05), which implied that high-risk patients were more sensitive to the other five drugs, while low-risk patients were more sensitive to methotrexate (Figure 10C).

### Construction and Evaluation of Nomogram

**FIGURE 11:**
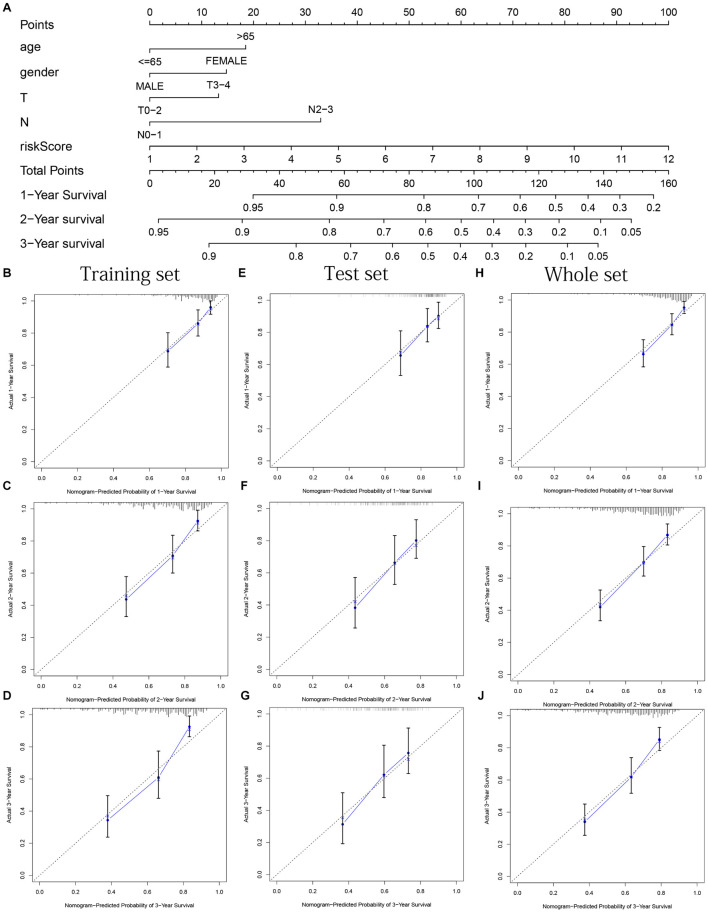
Nomogram based on prognostic model accurately predicts the OS of HNSCC patients. **(A)** The nomogram composed of various clinical risk factors and risk scores predicts the 1-, 2-, and 3-year survival probability of HNSCC patients. **(B–D)** One, 2, and 3 years internal calibration curve of the training set, respectively. **(E–G)** One, 2, and 3 years internal calibration curve of the test set, respectively. **(H–J)** One, 2, and 3 years internal calibration curve of the whole set, respectively.

**FIGURE 12:**
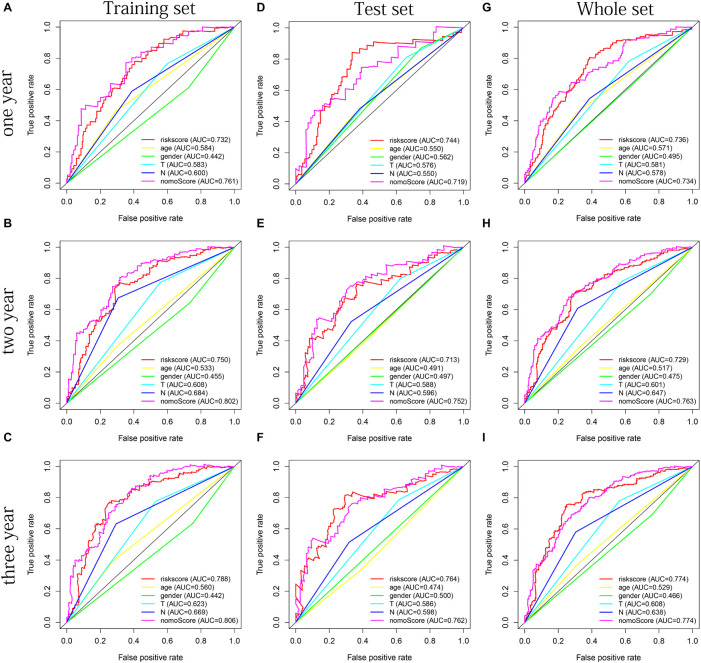
The multifactor ROC curves confirm the best predictive performance of nomogram. **(A–C)** One, 2, and 3 years of multifactor ROC curves in training set, respectively. **(D–F)** One, 2, and 3 years of multifactor ROC curves in the test set, respectively. **(G–I)** One, 2, and 3 years of multifactor ROC curves in the whole set, respectively.

Age, gender, T stage, N stage, and risk score were observed to be significantly related to OS by univariate cox regression analysis based on the training set (all *p* < 0.05, Figure 5J). From the nomogram constructed by these factors, we observed that risk score was the most important factor affecting patients’ survival, followed by N stage, age, gender, and T stage (Figure 11A). Through the calibration curve, we confirmed that the nomogram all had a good consistency between the predicted and the actual 1-, 2-, and 3-OS in training, test, and whole sets (training set, Figures 11B–D; test set, Figures 11E–G; whole set, Figures 11H–J). The ROC curves of all sets all confirmed that our nomogram not only had better predictive value (AUC values all >0.7) but also had better predictive ability of 1-, 2- and 3-year survival than other clinical factors (training set, Figures 12A–C; test set, Figures 12D–F; whole set, Figures 12G–I).

### Immunohistochemistry Staining

**FIGURE 13:**
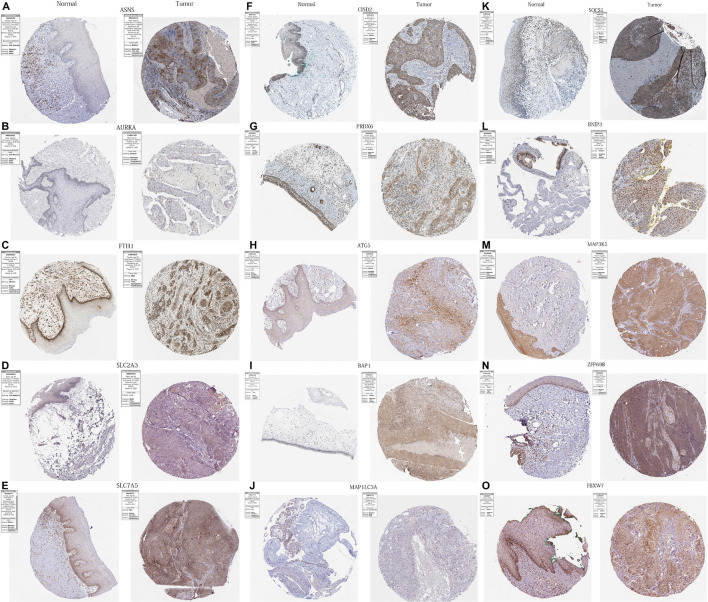
Immunohistochemistry images of 15 PR-DE-FRGs in HNSCC and normal tissues of the head and neck. **(A)** ASNS, **(B)** AURKA, **(C)** FTH1, **(D)** SLC2A3, **(E)** SLC7A5, **(F)** CISD2, **(G)** PRDX6, **(H)** ATG5, **(I)** BAP1, **(J)** MAP1LC3A, **(K)** SOCS1, **(L)** BNIP3, **(M)** MAP3K5, **(N)** ZFP69B, and **(O)** FBXW7.

We obtained immunohistochemical staining images of the protein expression of 15 PR-DE-FRGs (ASNS, AURKA, FTH1, SLC2A3, SLC7A5, CISD2, PRDX6, ATG5, BAP1, MAP1LC3A, SOCS1, BNIP3, MAP3K5, ZFP69B, and FBXW7) in HNSCC and normal tissues of the head and neck (Figure 13). By comparing the images of immunohistochemical staining in HNSCC tissue and normal tissues of the head and neck, we found that 11 DE-FRGs (ASNS, AURKA, FTH1, SLC2A3, SLC7A5, CISD2, PRDX6, ATG5, BAP1, MAP1LC3A, and SOCS1) showed differences in proteins expression (Figures 13A–K). The protein expression of ASNS, AURKA, FTH1, SLC2A3, SLC7A5, CISD2, ATG5, BAP1, and SOCS1 in the HNSCC tissue is higher than that in normal tissues of the head and neck (Figures 13A–F,H,I,K). On the contrary, the protein expression of PRDX6 and MAP1LC3A was observed to be lower in the HNSCC tissue (Figures 13G,J). All these support the results of the differential expression of these genes in our analysis. The images of the remaining four PR-DE-FRGs (BNIP3, MAP3K5, ZFP69B, and FBXW7) with no difference in protein expression are shown in Figures 13L–O.

## Discussion

In the research, we obtained 29 PR-DE-FRGs through difference and univariate cox regression analysis and screened 17 PR-DE-FRGs through LASSO regression to construct a prognostic model. The prognostic model showed good performance in all data randomly divided into three sets. Through the correlation analysis between the immune infiltrating cells and immune functions and the model, many meaningful results have been observed. Also, from the perspective of mutation, a series of associations between mutation and ferroptosis, even the development and prognosis of HNSCC have been initially established. In terms of clinical application, the prognostic model shows good distinguishing performance in the expression of ICIS gene and the sensitivity of commonly used chemotherapy drugs. Not only that, the combined model constructed by the prognostic model is also excellent in predicting the prognosis of HNSCC patients.

As we all know, different ferroptosis-related genes play different roles in ferroptosis. Among the 17 genes used to construct the prognostic model, 4 were identified as genes that inhibit ferroptosis (FTH1, CISD2, LINC00336, and PRDX6), 5 were identified as genes that drive ferroptosis (ATG5, BAP1, FBXW7, MAP1LC3A, and SOCS1), and 8 (ASNS, AURKA, BNIP3, DRD4 MAP3K5, SLC2A3, SLC7A5, and ZFP69B) were identified as genes that suggest ferroptosis in previous research. Past studies have found that ferroptosis can inhibit tumor growth and contribute to chemotherapy, immune checkpoint blockade, and radiotherapy, which have a positive effect on the prognosis of patients (Wang et al., 2019c; Lei et al., 2020; Luo et al., 2021). The role of these ferroptosis-related genes in the prognosis of HNSCC samples is also supported in our study. First, in our analysis, FTH1, CISD2, and PRDX6 were identified as risk factors, while BAP1, FBXW7, MAP1LC3A, and SOCS1 were identified as protective factors. In further survival analysis, it was also found that patients with high expression of BAP1, FBXW7, MAP1LC3A, and SOCS1 had a better prognosis, while patients with high expression of FTH1 and PRDX had a worse prognosis.

Except for PRDX6, FBXW7, and MAP1LC3A, the other 14 genes were upregulated in HNSCC. AURKA is abundantly expressed in a variety of cancer types (Chow et al., 2017) and plays an important role in the proliferation of ovarian cancer cells (Yuan and Huang, 2019). AURKA overexpression can also inhibit ferroptosis of cancer cells by inhibiting GPX4 (Gomaa et al., 2019). Not only that, the high expression of AURKA may also be a poor prognostic marker for adrenocortical carcinoma, renal clear cell carcinoma, hepatocellular carcinoma (HCC), lung adenocarcinoma (ADC), and mesothelioma (Du et al., 2021). BNIP3 can promote tumor growth by promoting necrosis and autophagy and is associated with poor prognosis of patients (Vakkila and Lotze, 2004; Rosenfeldt and Ryan, 2009; Petrova et al., 2015). BNIP3 has also been upregulated in lung cancer and is associated with poor prognosis (Petrova et al., 2015). It is generally believed that SLC7A5 is related to tumor development, angiogenesis, and poor prognosis of cancer patients as an ferroptosis regulator involved in energy metabolism (Giglia et al., 2014). Overexpression of CISD2 in head and neck cancer can enhance the resistance of sulfasalazine to ferroptosis (Kim et al., 2018). CISD2, which was found to be significantly upregulated in lung ADC samples, stimulates cancer cell proliferation and survival through elevated reactive oxygen species levels and ultimately leads to poor prognosis (Li et al., 2017). LINC00336 was found to be upregulated in lung cancer and acts as a competitive endogenous RNA to inhibit ferroptosis in lung cancer to induce tumor formation (Wang et al., 2019b). PRDX6 has the ability to remove lipid reactive oxygen species (LOOH) and inhibit ferroptosis (Lu et al., 2019). PRDX6 is overexpressed in a variety of cancers and is involved in the tumor progression of different tumors, such as lung cancer (Ho et al., 2010), thyroid (Nicolussi et al., 2014), and colorectal cancer (Huang et al., 2018). BAP1 induces ferroptosis by inhibiting cystine uptake and glutathione synthesis based on SLC7A11 inhibition, thereby inhibiting tumor development (Zhang et al., 2018, 2019). Renal cell carcinoma lacking BAP1 has also been found to be associated with a poor prognosis (Kapur et al., 2013). As a tumor suppressor in the process of human carcinogenesis, Fbxw7 is often observed to be downregulated in a variety of human cancers and is associated with a poor prognosis (Welcker and Clurman, 2008). SOCS1 can induce cells to be sensitive to ferroptosis by regulating the expression of p53 target genes (Saint-Germain et al., 2017). SOCS1, which was identified as a tumor suppressor gene for hepatocellular carcinoma (HCC), was found to be upregulated in hepatocytes and was associated with a good prognosis (Khan et al., 2020). The conclusions of these studies are unexpectedly consistent with our prognostic results. Currently, the role of ferroptosis in HNSCC is unclear. Although the function of many genes in cancer is still unclear, our research provides a new perspective on their potential role in HNSCC. Not only that, it can be found in the PPI and correlation network of these genes that they closely affect each other, which means that they may affect the progress of HNSCC through a complex interaction network.

Wang et al. (2019d) found that CD8 + T cells release cytokines to drive ferroptosis to kill tumor cells, including tumor necrosis factor and interferon γ; blocking them will eliminate the ferroptosis induced by T cells. Yee et al. (2020) also found that ferroptosis induced by neutrophils promoted tumor necrosis in the progression of glioblastoma. These studies have shown that the immune system can inhibit tumorigenesis in part by promoting ferroptosis of cancer cells. In the enrichment analysis of GSEA, it is found that our ferroptosis-related signatures may be involved in various immune-related BPs, CCs, and MFs, suggesting that ferroptosis is closely related to tumor immunity in HNSCC. For this reason, we further explored the interaction between tumor immunity and ferroptosis in HNSCC from the perspective of immune microenvironment including immune infiltrating cells and immune function. Costimulation was found to promote the proliferation and survival of CD8 + T cells and TRegs (Chen and Flies, 2013). Combining our results, we infer that the increased T cell costimulation in patients in the low-risk group may promote the proliferation of CD8 + T cells and TRegs, resulting in an increase in their content in HNSCC. The increased CD8 + T cells release interferon γ to increase the response of interferon γ to drive more ferroptosis to kill tumor cells, inhibit the progression of HNSCC, and ultimately lead to a better prognosis. TFH has been proven to play an antitumor immune cell effect by assisting CD8 effector T cells or directly eliminating tumor cells as cytotoxic T cells (Tran et al., 2014; Shi et al., 2018). Therefore, the higher TFH in HNSCC patients in the low-risk group may also induce ferroptosis by assisting CD8 effector T cells exerting antitumor effects. In breast cancer, mast cells are almost only found around the tumor and seem to have cytolytic activity on tumor cells (Rovere et al., 2007). Therefore, it is regarded as an antitumor factor and is associated with a good prognosis (Dabiri et al., 2004), which seems to explain the presence of more mast cells in HNSCC.

Gene mutations, especially TP53 mutations, which account for more than 50% of tumor mutations, play an important role in the occurrence and development of tumors. In our study, TP53 mutations were found to be widely present in 72% of HNSCC patients from CGA database. As a reflection of the number of mutations in tumor cells, the impact of TMB on the prognosis of tumor patients is still controversial. TMB is considered a poor prognostic marker in neuroblastoma, resectable pancreatic cancer, and diffuse glioma (Elisa et al., 2018; Hwang et al., 2018; Luo et al., 2020), while it is a good prognostic marker in non-small cell lung cancer (Devarakonda et al., 2018). In HNSCC, we found that the TMB of the high-risk group was significantly higher, which may explain that patients in the high-TMB group may have fewer ferroptosis and lead to a worse prognosis. In our study, it was found that the TP53 mutation group had a worse prognosis, which was also supported by many related studies. In many sporadic cancers, TP53 mutations have also been observed to be associated with poor prognosis (Kandoth et al., 2013). In addition to tumor suppressor function, TP53 can also mediate the process of ferroptosis and inhibit tumor growth by regulating cell cystine metabolism and ROS response (Le et al., 2017). In contrast, TP53 mutations can inhibit ferroptosis by altering cellular iron acquisition and metabolism, thereby promoting cancer progression (Thompson et al., 2020). We observed in HNSCC that patients in the TP53 mutation group had a higher risk score and were associated with a worse prognosis. This suggests that TP53 mutations in HNSCC may also inhibit ferroptosis, promote tumor progression, and lead to poor prognosis.

Keynote042, 024, and 021 based on pembrolizumab, Checkmate057 based on nivolumab, and IMpower131 and 150 based on atezulizumab all show that patients with high expression of PD-L1 can benefit more in immunotherapy. At present, the efficacy evaluation of PD-L1 detection for immunotherapy treatment of NSCLC has obtained the latest NCCN level 1 recommendation. In view of the conclusion that ferroptosis contributes to chemotherapy and immune checkpoint blocking therapy, we evaluated the possibility of prognostic models as markers for chemotherapy and immune checkpoint blocking treatment effect. From the corresponding results, it is found that our prognostic model can be used as expression value of PD-L1, PD-1, CTLA4, CD96, and TIGIT and six commonly used chemotherapeutic drug sensitivity prediction markers, which shows that our model may be used to predict the corresponding immunotherapy and chemotherapy efficacy to minimize adverse reactions.

As a statistical prediction model of cancer-specific survival probability, nomogram can combine the influence of several independent clinical variables to provide a personalized prognostic model for each patient (Duan et al., 2016). Due to the heterogeneity of cancer cells, patients will have different disease states at different times and lead to different clinical outcomes (Simson et al., 2007). Therefore, it is very necessary to construct a nomogram with high prediction accuracy to accurately predict the prognosis of HNSCC patients, which is conducive to precise treatment and improves the treatment benefits of patients. After multiple methods of verification, the nomogram formed by our risk score satisfies this requirement well.

There are many prognostic models focusing on HNSCC. She et al. (2020) screened 27 prognostic immune-related genes to construct a signature that can effectively predict the prognosis of patients (She et al., 2020). In this article, the researchers have initially explored the role that immunity may play in the progression of HNSCC through functional analysis. Lu et al. (2019) paid close attention to the relationship between tumor-associated macrophages and HNSCC. In this study, 10 prognostic-related differentially expressed marker genes of macrophage were screened to construct a model to predict the prognosis of HNSCC patients with higher sensitivity (Lu et al., 2019). The author not only discovered the key transcription factors that regulate marker genes of macrophages and their regulatory relationship but also found four areas related to tumor-associated macrophages function in HNSCC through GSEA enrichment analysis: intercellular matrix remodeling, tumor killing, metabolic reprogramming, and tumor immune-related pathways (Lu et al., 2019). Chen et al. (2021) adopted the novel weighted gene coexpression network analysis to identify 22 immune-related core genes and further screened 3 prognostic-related genes to construct a prognostic model. Not only that, this model shows good prognostic ability in both the training cohort and the external validation cohort. Our study focuses on the role of iron death in HNCC. This work not only used three sets to verify the ability of the model to predict the prognosis of patients but also explored the possible role of ferroptosis in the progression of HNSCC from the perspective of the immune microenvironment and TP53 mutation.

Although we have constructed a prognostic model with good clinical application value, and explored the role of ferroptosis in the development and prognosis of HNSCC from multiple perspectives such as immunity and mutation, and many valuable conclusions are obtained, there are still many shortcomings. First of all, because tobacco, HPV, and alcohol have a synergistic effect on HNSCC tumor progression, it seems essential to include tobacco and alcohol status in their research. However, it is difficult for us to obtain these data from the database and correct the influence of these factors. What is more, we could not find a dataset with both the transcriptome data of these 17 DE-FRG and survival data in other databases for external verification. To make up for this shortcoming, we split the HNSCC dataset of TCGA into three sets for multiple verification of the conclusions obtained. Second, although we used IHC images from clinical specimens from the HPA database to verify the differential expression of 11 PR-DE-FRGs at the protein level, the differential expression of 6 PR-DE-FRGs is still unknown. Due to various limitations of realistic conditions, it is difficult for us to collect clinical specimens for experiments to verify these results and conclusions. The good news is that our model performs well in all three data sets, but the reliability of the conclusions will still be questioned by future research results. Finally, some of the conclusions we got are only reasonable inferences based on the previous research conclusions and the results of our analysis. It only provides a new perspective for future research, and it is difficult to be treated as a conclusive conclusion.

## Data Availability Statement

The datasets presented in this study can be found in online repositories. The names of the repository/repositories and accession number(s) can be found in the article/supplementary material.

## Author Contributions

XF and LL designed the research. XF and YO analyzed the data. XF, HL, XZ, and MC prepared the figures. XF, LL, DY, LZ, and QL wrote and revised the manuscript. All authors contributed to the article and approved the submitted version.

## Conflict of Interest

The authors declare that the research was conducted in the absence of any commercial or financial relationships that could be construed as a potential conflict of interest.

## Publisher’s Note

All claims expressed in this article are solely those of the authors and do not necessarily represent those of their affiliated organizations, or those of the publisher, the editors and the reviewers. Any product that may be evaluated in this article, or claim that may be made by its manufacturer, is not guaranteed or endorsed by the publisher.

## Footnotes

1
cancergenome.nih.gov

2
zhounan.org/ferrdb

3
timer.comp-genomics.org

4
tcia.at/home

5
proteinatlas.org

## References

[B1] Bray F. Ferlay J. Soerjomataram I. Siegel R. L. Torre L. A. Jemal A. (2018). Global cancer statistics 2018: GLOBOCAN estimates of incidence and mortality worldwide for 36 cancers in 185 countries. *CA Cancer J. Clin.* 68 394–424. 10.3322/caac.21492 30207593

[B2] Cao D. Xu H. Xu X. Guo T. Ge W. (2019). High tumor mutation burden predicts better efficacy of immunotherapy: a pooled analysis of 103078 cancer patients. *Oncoimmunology* 8:e1629258. 10.1080/2162402x.2019.1629258 31428527 PMC6685508

[B3] Chen L. Flies D. B. (2013). Molecular mechanisms of T cell co-stimulation and co-inhibition. *Nat. Rev. Immunol.* 13 227–242. 10.1038/nri3405 23470321 PMC3786574

[B4] Chen Y. Li Z. Y. Zhou G. Q. Sun Y. (2021). An immune-related gene prognostic index for head and neck squamous cell carcinoma. *Clin. Cancer Res.* 27 330–341. 10.1158/1078-0432.ccr-20-2166 33097495

[B5] Chow Y. Alias H. Jamal R. (2017). Meta-analysis of gene expression in relapsed childhood B-acute lymphoblastic leukemia. *BMC Cancer* 17:120. 10.1186/s12885-017-3103-1 28183295 PMC5301337

[B6] Cohen E. Bell R. B. Bifulco C. B. Burtness B. Ferris R. L. (2019). The Society for Immunotherapy of Cancer consensus statement on immunotherapy for the treatment of squamous cell carcinoma of the head and neck (HNSCC). *J. Immunother. Cancer* 7:184. 10.1186/s40425-019-0662-5 PMC6632213 31307547

[B7] Cui J. Zheng L. Zhang Y. Xue M. (2021). Bioinformatics analysis of DNMT1 expression and its role in head and neck squamous cell carcinoma prognosis. *Sci. Rep.* 11:2267. 10.1038/s41598-021-81971-5 33500531 PMC7838186

[B8] Dabiri S. Huntsman D. Makretsov N. Cheang M. Gilks B. Badjik C. (2004). The presence of stromal mast cells identifies a subset of invasive breast cancers with a favorable prognosis. *Mod. Pathol.* 17 690–695. 10.1038/modpathol.3800094 15044916

[B9] Devarakonda S. Rotolo F. Tsao M. Lanc I. Brambilla E. Masood A. (2018). Tumor mutation burden as a biomarker in resected non-small-cell Lung Cancer. *J. Clini. Oncol.* 36 2995–3006. 10.1200/jco.2018.78.1963 30106638 PMC6804865

[B10] Du R. Huang C. Liu K. Li X. Dong Z. (2021). Targeting AURKA in Cancer: molecular mechanisms and opportunities for Cancer therapy. *Mol. Cancer* 20:15. 10.1186/s12943-020-01305-3 33451333 PMC7809767

[B11] Duan J. Deng T. Ying G. Huang D. Zhang H. Zhou L. (2016). Prognostic nomogram for previously untreated patients with esophageal squamous cell carcinoma after esophagectomy followed by adjuvant chemotherapy. *Jpn. J. Clin. Oncol.* 46 336–343. 10.1093/jjco/hyv206 26819278 PMC4886130

[B12] Elisa G. Sandra D. Annalisa A. Giuseppe T. Valentina I. Eva F. (2018). Mutational burden of resectable pancreatic cancer, as determined by whole transcriptome and whole exome sequencing, predicts a poor prognosis. *Int. J. Oncol.* 52 1972–1980. 29620163 10.3892/ijo.2018.4344

[B13] Fang X. N. Miao Y. Hua L. Cheng L. Cong X. Yang G. W. (2018). Comprehensive analysis of competitive endogenous RNAs network associated with head and neck squamous cell carcinoma. *Sci. Rep.* 8 10544–10568. 30002503 10.1038/s41598-018-28957-y PMC6043529

[B14] Gao J. Kwan P. W. Shi D. (2010). Sparse kernel learning with LASSO and bayesian inference algorithm. *Neural Netw.* 23 257–264. 10.1016/j.neunet.2009.07.001 19604671

[B15] Geeleher P. Cox N. J. Huang R. (2014). Clinical drug response can be predicted using baseline gene expression levels and in vitro drug sensitivity in cell lines. *Genome Biol.* 15:R47. 10.1186/gb-2014-15-3-r47 PMC4054092 24580837

[B16] Giglia J. White M. Hart A. Toro J. Freytes C. Holt C. (2014). A single nucleotide polymorphism in SLC7A5 is associated with gastrointestinal toxicity after high-dose melphalan and autologous stem cell transplantation for multiple myeloma. *Biol. Blood Marrow Trans.* 20 1014–1020. 10.1016/j.bbmt.2014.03.022 24704384 PMC4076151

[B17] Gomaa A. Peng D. Chen Z. Soutto M. Abouelezz K. Corvalan A. (2019). Epigenetic regulation of AURKA by miR-4715-3p in upper gastrointestinal cancers. *Sci. Rep.* 9:16970. 10.1038/s41598-019-53174-6 31740746 PMC6861278

[B18] He X. Xu H. Zhao W. Zhan M. Li Y. Liu H. (2019). POPDC3 is a potential biomarker for prognosis and radioresistance in patients with head and neck squamous cell carcinoma. *Oncol. Lett.* 18 5468–5480. 10.3892/ol.2019.10888 31612055 PMC6781657

[B19] Ho J. Lee S. Lee S. Yoon S. Kang G. Hwang S. (2010). Phospholipase A2 activity of peroxiredoxin 6 promotes invasion and metastasis of lung cancer cells. *Mol. Cancer Ther.* 9 825–832. 10.1158/1535-7163.Mct-09-0904 20354123

[B20] Huang W. Huang C. Hsieh M. Kuo Y. Tung S. Shen C. (2018). Expression of PRDX6 correlates with migration and invasiveness of colorectal cancer cells. *Cell. Physiol. Biochem.* 51 2616–2630. 10.1159/000495934 30562740

[B21] Hwang W. L. Wolfson R. L. Andrzej N. Marcus K. J. Dubois S. G. Daphne H. K. (2018). Clinical impact of tumor mutational burden in neuroblastoma. *J. Natl. Cancer Inst.* 111 695–699. 10.1093/jnci/djy157 30307503 PMC6624164

[B22] Jiang L. Kon N. Li T. Wang S. Su T. Hibshoosh H. (2015). Ferroptosis as a p53-mediated activity during tumour suppression. *Nature* 520 57–62. 10.1038/nature14344 25799988 PMC4455927

[B23] Kandoth C. McLellan M. Vandin F. Ye K. Niu B. Lu C. (2013). Mutational landscape and significance across 12 major cancer types. *Nature* 502 333–339. 10.1038/nature12634 24132290 PMC3927368

[B24] Kapur P. Peña-Llopis S. Christie A. Zhrebker L. Pavía-Jiménez A. Rathmell W. K. (2013). Effects on survival of BAP1 and PBRM1 mutations in sporadic clear-cell renal-cell carcinoma: a retrospective analysis with independent validation. *Lancet Oncol.* 14 159–167. 10.1016/s1470-2045(12)70584-3 23333114 PMC4674067

[B25] Khan M. Ghosh A. Variya B. Santharam M. A. Ilangumaran S. (2020). Prognostic significance of SOCS1 and SOCS3 tumor suppressors and oncogenic signaling pathway genes in hepatocellular carcinoma. *BMC Cancer* 20:774. 10.1186/s12885-020-07285-3 32807134 PMC7433106

[B26] Kim E. Shin D. Lee J. Jung A. Roh J. (2018). CISD2 inhibition overcomes resistance to sulfasalazine-induced ferroptotic cell death in head and neck cancer. *Cancer Lett.* 432 180–190. 10.1016/j.canlet.2018.06.018 29928961

[B27] Koboldt D. C. Chen K. Wylie T. Larson D. E. McLellan M. D. Mardis E. R. (2009). VarScan: variant detection in massively parallel sequencing of individual and pooled samples. *Bioinformatics* 25 2283–2285. 10.1093/bioinformatics/btp373 19542151 PMC2734323

[B28] Le J. Ning K. Li T. Wang S. J. Tao S. Hibshoosh H. (2017). Ferroptosis as a p53-mediated activity during tumour suppression. *Nature* 520, 57–62. 10.1038/nature14344 25799988 PMC4455927

[B29] Lei G. Zhang Y. Koppula P. Liu X. Zhang J. Lin S. H. (2020). The role of ferroptosis in ionizing radiation-induced cell death and tumor suppression. *Cell Res.* 30 146–162. 10.1038/s41422-019-0263-3 31949285 PMC7015061

[B30] Li S. Chen C. Chen Y. Yen Y. Fang W. Tsai F. (2017). Upregulation of CISD2 augments ROS homeostasis and contributes to tumorigenesis and poor prognosis of lung adenocarcinoma. *Sci. Rep.* 7:11893. 10.1038/s41598-017-12131-x 28928421 PMC5605537

[B31] Liang C. Zhang X. Yang M. Dong X. (2019). Recent progress in ferroptosis inducers for cancer therapy. *Adv. Mater.* 31:e1904197. 10.1002/adma.201904197 31595562

[B32] Liu J. Meng H. Nie S. Sun Y. Jiang P. Li S. (2020). Identification of a prognostic signature of epithelial ovarian cancer based on tumor immune microenvironment exploration. *Genomics* 112 4827–4841. 10.1016/j.ygeno.2020.08.027 32890701

[B33] Lu B. Chen X. Hong Y. Zhu H. He Q. Yang B. (2019). Identification of PRDX6 as a regulator of ferroptosis. *Acta Pharmacol. Sin.* 40 1334–1342. 10.1038/s41401-019-0233-9 31036877 PMC6786318

[B34] Luo T. Wang L. Ge J. Lan Y. Xu Y. (2020). Tumor mutational burden is associated with poor outcomes in diffuse glioma. *BMC Cancer* 20:213. 10.1186/s12885-020-6658-1 32164609 PMC7069200

[B35] Luo Y. Huang Q. He B. Liu Y. Huang S. Xiao J. (2021). Regulation of ferroptosis by non-coding RNAs in the development and treatment of cancer (Review). *Oncol. Rep.* 45 29–48. 10.3892/or.2020.7836 33155665 PMC7709825

[B36] Mantovani F. Collavin L. Del Sal G. (2019). Mutant p53 as a guardian of the cancer cell. *Cell Death Differ.* 26 199–212. 10.1038/s41418-018-0246-9 30538286 PMC6329812

[B37] Nicolussi A. D’Inzeo S. Mincione G. Buffone A. Di Marcantonio M. Cotellese R. (2014). PRDX1 and PRDX6 are repressed in papillary thyroid carcinomas via BRAF V600E-dependent and -independent mechanisms. *Int. J. Oncol.* 44 548–556. 10.3892/ijo.2013.2208 24316730

[B38] Petrova V. Mancini M. Agostini M. Knight R. Annicchiarico-Petruzzelli M. Barlev N. (2015). TAp73 transcriptionally represses BNIP3 expression. *Cell Cycle* 14 2484–2493. 10.1080/15384101.2015.1044178 25950386 PMC4612661

[B39] Quan H. Yan L. Wang S. Wang S. (2019). Clinical relevance and significance of programmed death-ligand 1 expression, tumor-infiltrating lymphocytes, and p16 status in sinonasal squamous cell carcinoma. *Cancer Manag. Res.* 11 4335–4345. 10.2147/cmar.S201568 31190998 PMC6514258

[B40] Rosenfeldt M. T. Ryan K. M. (2009). The role of autophagy in tumour development and cancer therapy. *Expert Rev. Mol. Med.* 11:e36. 10.1017/S1462399409001306 PMC2811398 19951459

[B41] Rovere F. D. Granata A. Familiari D. D’Arrigo G. Basile G. (2007). Mast cells in invasive ductal breast cancer: different behavior in high and minimum hormone-receptive cancers. *Anticancer Res.* 27 2465–2471. 17695540

[B42] Saint-Germain E. Lian M. Vernier M. Bobbala D. Ferbeyre G. (2017). SOCS1 regulates senescence and ferroptosis by modulating the expression of p53 target genes. *Aging* 9 2137–2162. 10.18632/aging.101306 29081404 PMC5680560

[B43] Samstein R. Lee C. Shoushtari A. Hellmann M. Shen R. Janjigian Y. (2019). Tumor mutational load predicts survival after immunotherapy across multiple cancer types. *Nat. Genet.* 51 202–206. 10.1038/s41588-018-0312-8 30643254 PMC6365097

[B44] She Y. Kong X. Ge Y. Yin P. Fang S. (2020). Immune-related gene signature for predicting the prognosis of head and neck squamous cell carcinoma. *Cancer Cell Int.* 20:22. 10.1186/s12935-020-1104-7 PMC6969412 31988638

[B45] Shi W. Dong L. Sun Q. Ding H. Meng J. Dai G. (2018). Follicular helper T cells promote the effector functions of CD8+ T cells via the provision of IL-21, which is downregulated due to PD-1/PD-L1-mediated suppression in colorectal cancer. *Exp. Cell Res.* 372 35–42. 10.1016/j.yexcr.2018.09.006 30205088

[B46] Simson L. Ellyard J. I. Dent L. A. Matthaei K. I. Rothenberg M. E. Foster P. S. (2007). Regulation of carcinogenesis by IL-5 and CCL11: a potential role for eosinophils in tumor immune surveillance. *J. Immunol.* 178 4222–4229. 10.4049/jimmunol.178.7.4222 17371978

[B47] Tada H. Takahashi H. Kawabata-Iwakawa R. Nagata Y. Uchida M. Shino M. (2020). Molecular phenotypes of circulating tumor cells and efficacy of nivolumab treatment in patients with head and neck squamous cell carcinoma. *Sci. Rep.* 10:21573. 10.1038/s41598-020-78741-0 33299117 PMC7726556

[B48] Thompson L. Oliveira T. Hermann E. Chowanadisai W. Clarke S. Montgomery M. (2020). Distinct TP53 mutation types exhibit increased sensitivity to ferroptosis independently of changes in iron regulatory protein activity. *Int. J. Mol. Sci.* 21:6751. 10.3390/ijms21186751 32942535 PMC7555626

[B49] Tran E. Turcotte S. Gros A. Robbins P. F. Lu Y. C. Dudley M. E. (2014). Cancer immunotherapy based on mutation-specific CD4+ T cells in a patient with epithelial cancer. *Science* 344 641–645. 10.1126/science.1251102 24812403 PMC6686185

[B50] Vakkila J. Lotze M. T. (2004). Inflammation and necrosis promote tumour growth. *Nat. Rev. Immunol.* 4 641–648. 10.1038/nri1415 15286730

[B51] Wang H. Mustafa A. Liu S. Liu J. Lv D. Yang H. (2019a). Immune checkpoint inhibitor toxicity in head and neck cancer: from identification to management. *Front. Pharmacol.* 10:1254. 10.3389/fphar.2019.01254 31708780 PMC6819434

[B52] Wang M. Mao C. Ouyang L. Liu Y. Lai W. Liu N. (2019b). Long noncoding RNA LINC00336 inhibits ferroptosis in lung cancer by functioning as a competing endogenous RNA. *Cell Death Differ.* 26 2329–2343. 10.1038/s41418-019-0304-y 30787392 PMC6889193

[B53] Wang W. Green M. Choi J. Gijón M. Kennedy P. Johnson J. (2019c). CD8 T cells regulate tumour ferroptosis during cancer immunotherapy. *Nature* 569 270–274. 10.1038/s41586-019-1170-y 31043744 PMC6533917

[B54] Wang W. Green M. Choi J. E. Gijón M. Zou W. (2019d). CD8+ T cells regulate tumour ferroptosis during cancer immunotherapy. *Nature* 569 1–5. 10.1038/s41586-019-1170-y PMC6533917 31043744

[B55] Welcker M. Clurman B. E. (2008). FBW7 ubiquitin ligase: a tumour suppressor at the crossroads of cell division, growth and differentiation. *Nat. Rev. Cancer* 8 83–93. 10.1038/nrc2290 18094723

[B56] Wu Y. Xu J. Du C. Wu Y. Xia D. Lv W. (2019). The predictive value of tumor mutation burden on efficacy of immune checkpoint inhibitors in cancers: a systematic review and meta-analysis. *Front. Oncol.* 9:1161. 10.3389/fonc.2019.01161 31750249 PMC6848266

[B57] Xia Y. Liu S. Li C. Ai Z. Shen W. Ren W. (2020). Discovery of a novel ferroptosis inducer-talaroconvolutin A-killing colorectal cancer cells in vitro and in vivo. *Cell Death Dis.* 11:988. 10.1038/s41419-020-03194-2 33203867 PMC7673992

[B58] Yee P. P. Wei Y. Kim S. Y. Lu T. Li W. (2020). Neutrophil-induced ferroptosis promotes tumor necrosis in glioblastoma progression. *Nat. Commun.* 11:5424. 10.1038/s41467-020-19193-y PMC7591536 33110073

[B59] Yi J. Zhu J. Wu J. Thompson C. Jiang X. (2020). Oncogenic activation of PI3K-AKT-mTOR signaling suppresses ferroptosis via SREBP-mediated lipogenesis. *Proc. Natl. Acad. Sci. U.S.A.* 117 31189–31197. 10.1073/pnas.2017152117 33229547 PMC7733797

[B60] Yi L. Wu G. Guo L. Zou X. Huang P. (2020). Comprehensive analysis of the PD-L1 and immune infiltrates of mA RNA methylation regulators in head and neck squamous cell carcinoma. *Mol. Ther. Nucleic Acids* 21 299–314. 10.1016/j.omtn.2020.06.001 32622331 PMC7332506

[B61] Yuan L. Huang D. (2019). A network-guided association mapping approach from DNA methylation to disease. *Sci. Rep.* 9:5601. 10.1038/s41598-019-42010-6 30944378 PMC6447594

[B62] Zhang Y. Shi J. Liu X. Feng L. Gong Z. Koppula P. (2018). BAP1 links metabolic regulation of ferroptosis to tumour suppression. *Nat. Cell Biol.* 20 1181–1192. 10.1038/s41556-018-0178-0 30202049 PMC6170713

[B63] Zhang Y. Zhuang L. Gan B. (2019). BAP1 suppresses tumor development by inducing ferroptosis upon SLC7A11 repression. *Mol. Cell. Oncol.* 6:1536845. 10.1080/23723556.2018.1536845 30788415 PMC6370386

